# Diabetic sarcopenia in clinical practice: expert consensus and usability evaluation of a novel screening and diagnostic algorithm

**DOI:** 10.3389/fnut.2026.1906664

**Published:** 2026-10-07

**Authors:** Alejandro Sanz-Paris, Francisco José Tarazona-Santabalbina, Juana Carretero Gómez, Daniel de Luis Román, José Manuel García-Almeida, Fernando Garrachón-Vallo, Juan José López-Gómez, Germán Guzmán-Rolo, Pedro Pablo García-Luna

**Affiliations:** 1 Endocrinology Department, University Hospital Miguel Servet, Zaragoza, Spain; 2 Geriatrics Department, Hospital La Pedrera, Dénia, Spain; 3 FRAGIVAL Associated Clinical Group to CIBER de Fragilidad y Envejecimiento Saludable (CIBERFES) ISCIII, Madrid, Spain; 4 Medical School, Universidad Católica de Valencia San Vicente Mártir, Valencia, Spain; 5 Internal Medicine Department, University Hospital of Badajoz, Badajoz, Spain; 6 Endocrinology and Nutrition Service, Hospital Clínico Universitario de Valladolid and Valladolid Health Research Institute (IBioVALL), Valladolid, Spain; 7 Endocrinology and Nutrition Research Centre Faculty of Medicine University of Valladolid, Valladolid, Spain; 8 Center for Biomedical Research Network (CIBEROBN) of Obesity and Nutrition, Instituto de Salud Carlos III, Madrid, Spain; 9 Clinical Management Unit of Endocrinology and Nutrition, Virgen de la Victoria Clinical Hospital, Málaga, Spain; 10 Endocrinology and Nutrition Department, Hospital Virgen de la Victoria de Malaga, Malaga Biomedical Research Institute and BIONAND Platform, Malaga, Spain; 11 Department of Endocrinology and Nutrition, Quironsalud Malaga Hospital, Malaga, Spain; 12 Spanish Biomedical Research Center in Physiopathology of Obesity and Nutrition (CIBERObn), Carlos III Health Institute, Madrid, Spain; 13 Internal Medicine Department, Virgen Macarena University Hospital, Seville, Spain; 14 Medical Department, Abbott Laboratories, Madrid, Spain; 15 Endocrinology and Nutrition Clinical Management Unit, Institute of Biomedicine of Seville (IBiS), Hospital Universitario Virgen del Rocío/CSIC/University of Seville, Seville, Spain

**Keywords:** clinical algorithm, clinical usability, Delphi consensus, diabetic sarcopenia, sarcopenia screening, type 2 diabetes

## Abstract

**Introduction:**

Recently, an expert panel developed an algorithm for the screening, diagnosis, and staging of diabetic sarcopenia, a clinically relevant condition in the progression of diabetes. Sarcopenia is associated with an increased risk of falls, physical disability, reduced quality of life, and increased mortality.

**Methods:**

A qualitative study, with quantitative aspects, was conducted to assess medical specialists’ opinions on the applicability of the proposed algorithm. Data was collected using a questionnaire that included information relating to user experience, a usability questionnaire, a survey of healthcare professionals’ opinions and a consensus form administered using the Delphi method.

**Results:**

The questionnaire was completed by 299 doctors (65.9% were women, mean age 39.1 (SD 8.5) years and with a mean of 10.4 (SD 8) years’ experience). Most respondents indicated that screening for sarcopenia should be prioritised in patients with recent hospitalisations, major surgery, prolonged immobilisation and malignancies (Median 9, IQ0) and should be repeated annually in high-risk patients without a confirmed diagnosis (Median 8, IQ2). The algorithm was considered suitable for muscle assessment in people with diabetes. 90% of participants considered the tool to be easy to use, requiring no specialist staff (70%), and taking less than 7 min to apply (88%). For most respondents, use of the algorithm has changed the approach to decision-making regarding sarcopenia in patients with type 2 diabetes.

**Conclusion:**

The findings highlight the importance of systematic screening, diagnosis, and staging of diabetic sarcopenia, and demonstrate high levels of perceived applicability and usability of the proposed algorithm in routine clinical practice.

## Introduction

Sarcopenia is a progressive and widespread disorder associated with a reduction in the quantity and quality of muscle, as well as of its function (1, 2). This reduction results in an increased incidence of falls, fractures and physical limitations, reduced function, loss of quality of life, and increased mortality (1, 2).

The hallmark of diabetic sarcopenia is muscle mass atrophy characteristic of people with diabetes in contrast to the histological and physiological normality of muscle mass (MM) (3, 4). Diabetes behaves as an independent factor for major loss of skeletal muscle mass (5). In people with type 2 diabetes mellitus (T2DM), various changes occur in body composition, particularly in the form of increased visceral fat and decreased muscle and bone mass (6, 7) because of the metabolic dysfunction caused by diabetes (8).

Patients with diabetes mellitus have a high prevalence of sarcopenia, and in T2DM it is estimated to affect between 7 and 29.3% of all patients (9). Notably, this condition exacerbates the metabolic disorder and compromises the response to treatment (10). The reduction in muscle mass causes an increased risk of falls, fractures and physical disability, loss of quality of life and increased mortality (1, 2). Diabetic sarcopenia associated with frailty and complications of diabetes, is emerging as a significant category of complications leading to disability and dependency (3).

However, there are few references in the national and international guidelines to the measurement, diagnosis, and management of diabetic sarcopenia (DS), defined as a disorder characterised by the presence of type 1 or 2 diabetes mellitus and sarcopenia (3). A screening, diagnosis, and staging algorithm for diabetic sarcopenia has recently been described by a multidisciplinary panel of experts (11). The algorithm is intended to serve as a general-purpose tool for healthcare professionals treating patients with diabetes. It is designed to be practical and accessible, enabling the early and accurate identification of patients at risk of DS and facilitating interventions to improve clinical outcomes and quality of life. These protocols, as well as the operational definitions developed and the inclusion of sarcopenia in the International Classification of Diseases 10th revision Clinical Modification (ICD-10-CM), should encourage clinicians to diagnose and treat sarcopenia and DS in the elderly and those with diabetes (12).

The aim of this consensus study project was to evaluate the usability of the proposed algorithm under real-world clinical conditions, as well as to reach a consensus on different aspects related to the management of DS among specialists who routinely care for patients with diabetes. The study was structured in two complementary phases. In the first phase, the Delphi method was applied to validate the screening, diagnosis, staging and treatment criteria proposed by the expert panel, thus providing multidisciplinary support to the recommendations. In the second phase, the clinical applicability and usability of the algorithm were evaluated using standardised indicators of efficiency and satisfaction. This comprehensive approach aims not only to contribute to standardisation of the diagnosis of diabetic sarcopenia, but also to ensure that the algorithm serves as a flexible and effective tool that facilitates clinical decision-making and contributes to improving patient quality of life in clinical practice.

## Methods

### Study design

A qualitative study incorporating quantitative elements was conducted, the main objectives of which were: (a) to establish consensus on areas related to the practical application of the algorithm in various situations (Annex 1) and (b) to assess the clinical usability of the proposed algorithm for screening, diagnosis, and staging of diabetic sarcopenia.

### Coordinating group and preparation of the questionnaire

The coordinating group was responsible for proposing, defining and addressing existing challenges relating to specific aspects of the diabetes and sarcopenia algorithm, thereby providing the basis for formulating the Delphi questionnaire items. The group comprised specialists with extensive training in diabetes and clinical nutrition, including endocrinologists and internists. Two online meetings were held to define 16 consensus questions consensus study addressing screening criteria and indications, priority populations and risk factors, frequency and follow-up, diagnosis, staging and tools, and finally intervention and therapeutic management.

### Selection of the participants

Participation in the consensus study was voluntary, and the responses were coded to maintain anonymity and confidentiality. Specialist doctors in endocrinology and nutrition, geriatrics, internal medicine and oncology were selected, with the following characteristics: Active daily clinical practice, 3 years or more of experience in their speciality and an interest in nutrition and/or at least one scientific publication in the field of nutrition within the past 5 years. The participants were representatively distributed across Spain and a document explaining the consensus study and the survey was sent to them. All participants had used the algorithm on at least 5 occasions prior to the survey. No patient data have been recorded in the project.

### Part 1: consensus questionnaire (Delphi method)

The Delphi method was applied for evaluation and usability validation of the algorithm statements. The Delphi method is a structured process consisting of two consecutive rounds of questionnaires, widely used in obtaining results in clinical settings with a high degree of uncertainty. It aims to achieve consensus by systematically collecting expert opinions (13, 14).

In the first round, the participants were sent a document explaining the purpose of the study, the Delphi method, and how to complete the survey. All participants were asked to rate each question.

In the first round, the participants were asked to indicate their level of agreement with each statement using a nine-point scale (1: “strongly disagree” to 9: “strongly agree”). Scores of 1 to 3 (range 1) were considered to indicate a low degree of agreement, implementation or knowledge, depending on the question; scores of 4 to 6 (range 2) were considered uncertain; and scores of 7 to 9 (range 3) were considered to indicate a high degree of agreement. The first round remained open for 12 weeks, with a 4-week reminder sent to those specialists who had partially or totally completed the questionnaire or who had not done so. At the end of the first round, all participants had completed the survey, and their responses were coded and anonymised in a database.

For the analysis, descriptive statistics were used to summarise the results for each question, including the percentage of participants who scored between 1 and 9. Each statement that achieved a consensus of agreement was defined as having a median consensus score (MED) of ≥ 7 and an interquartile range (IQR) of ≤3. Likewise, a MED result of ≤3 was regarded as consensus to reject the statement, whilst an IQR of ≥ 4 or a MED of 4–6 was regarded as indicating no agreement. The statements without consensus were to be reviewed and included in a second round with only two possible responses (Yes/No), in which consensus would be considered when 50% or more of the responses were “Yes” or “No”.

Although the Delphi method is usually conducted in several rounds to refine consensus, a second round was not implemented in this study due to the high level of consensus achieved in the initial evaluation of the statements during the first round.

### Part 2: evaluation of applicability and usability

The questionnaire included a section specifically designed to measure healthcare professionals’ experience of using the algorithm, employing the System Usability Scale (SUS) and the Net Promoter Score (NPS), as well as questions regarding the existence of a nutritional screening routine prior to using the algorithm in this patient group, the average time taken to use the algorithm with their patients, and whether using the malnutrition and DS algorithm has changed their approach to clinical decision-making regarding nutrition and sarcopenia for their patients with T2DM.

The System Usability Scale (SUS) is a quick and agile method for assessing the usability of any system (15). This method jointly measures three key parameters in the interaction with the system: efficacy, efficiency and satisfaction. It consists of a 10-item questionnaire with responses on a 5-point Likert-scale ranging from “strongly disagree” to “strongly agree,” and affords a quantitative measure of the level of usability to be obtained with a single final score ranging from 0 to 100. A score above 68 is considered good and above 80 very good.

In addition, the NPS was calculated as an index to measure loyalty and satisfaction with the algorithm (16), based on the likelihood that users would recommend it to other colleagues or friends. The NPS is based on one question: “How likely are you to recommend the product or service to a family member or friend?” [Implicit mention in NPS methodology]. To respond, participants were asked to rate their likelihood on a scale of 0 to 10, where 0 means “Very unlikely” and 10 means “‘I would definitely recommend it”. Based on the assigned score, the specialists were classified into three groups: promoters (9 or 10 points), passives or indifferent (7 or 8 points) and detractors (≤6 points). To obtain the NPS index, the percentage of detractors was subtracted from the percentage of promoters. This result was then divided by the total responses obtained and multiplied by one hundred (100). This numerical indicator does not represent a percentage, but rather the level of user loyalty. The NPS index can range from −100 (if all are detractors) to 100 (if all are promoters). An NPS greater than 0 is perceived as good, and a score of 50 is considered excellent.

In addition to this, the average duration of use in their patients and clinical decision-making regarding nutrition and sarcopenia for patients were included.

### Data analysis

The statistical analysis of the data obtained was performed using Statistical Analysis System (SAS) software, version 9.4 (Cary, NC, United States).

For each statement assessed, the median consensus score (MED), the interquartile range (IQR) and the standard deviation were calculated. Descriptive statistics were used to summarise the results for each question, detailing the percentage of participants who assigned each score from 1 to 9. For comparisons involving more than two specialties (e.g., age or SUS scores), the Kruskal-Wallis test was used to assess overall differences among the five specialty groups. The non-parametric Mann–Whitney U test was reserved for post-hoc pairwise comparisons between specific groups where necessary. Additionally, Fisher’s exact test was employed to analyze categorical variables across the various specialties. Due to the descriptive and consensus-based nature of the study, no formal sample size calculation or power justification was performed, and the analysis focused on identifying professional trends and agreement levels. A *p*-value < 0.05 indicating a statistically significant difference.

## Results

### Participants

A total of 299 doctors took part, mainly specialists in endocrinology and nutrition, internal medicine and geriatrics, and internists (> 85%). The mean age was 39 (SD 8.5) years, with no statistically significant differences observed across specialities. The mean number of years of professional experience was 10.4 (SD 8.0). Table 1 shows the characteristics of the participants in the consensus study.

**Table 1:** Characteristics of the consensus study participants.

Sex	*N*	%
Male	102	34.11
Female	197	65.89
Speciality	*N*	%
Endocrinology	132	44.15
Internal medicine	94	31.44
Geriatrics	43	14.38
Oncology	15	5.02
Others	15	5.02
Speciality	Mean (SD)	
Years	39.11	8.52
Years of experience^*^	Mean (SD)	
Years	10.4	7.98

^*^Years of experience practising in their speciality (following their residency or speciality training).

### Characterisation questionnaire

For the nutritional diagnosis of the patients, 77.2% of those surveyed used the GLIM criteria (17), 17.3% the MNA method (18), 1.6% the SGA (19), and 3.6% other systems (Table 2). The GLIM criteria were more commonly used by endocrinologists (93.9%), while geriatricians and internists used them less frequently (62.7 and 60.6%, respectively).

**Table 2:** Method used by the participants in nutritional diagnosis.

Speciality	GLIM		SGA		MNA		Other	Total	
	*N*	%	*N*	%	*N*	%	*N*	*N*	%
Endocrinology	124	93.94	2	1.5	1	0.76	5	132	100
Geriatrics	27	62.79	.	0	14	32.6	2	43	100
Internal medicine	57	60.64	1	1.1	34	36.2	2	94	100
Oncology	12	80	1	6.7	1	6.67	1	15	100
Others	11	73.33	1	6.7	2	13.3	1	15	100
Total	231	77.26	5	1.7	52	17.4	11	299	100

Variable	Test	*P*-value
Speciality	Fisher	0.005

GLIM: Global Leadership Initiative on Malnutrition; SGA: Subjective Global Assessment; MNA: Mini-Nutritional Assessment.

The most used muscle function test in these patients was dynamometry (50.5%), followed by the Timed Up and Go (TUG) (20) test (17.7%). The chair test (21) was used by 16.6%, the Short Physical Performance Battery (SPPB) (22) in 10.7%, the gait speed test in 4.0%, and other methods in 0.6% (Table 3). Statistically significant differences were found across specialities, with endocrinology being the speciality that makes the most use of dynamometry (75%), whilst geriatrics is the one that makes the most use of the SPPB (51.1%).

**Table 3:** Functional tests used in the diagnosis of muscle function.

Speciality	Dynam		TUG		Chair		SPPB		Gait		Other		Total	
	*N*	%	*N*	%	*N*	%	*N*	%	*N*	%	*N*	%	*N*	%
Endocrinology	99	75	14	10.6	16	12.1	2	1.52	.	0	1	0.8	132	100
Geriatrics	8	18.6	4	9.3	3	6.98	22	51.2	6	14	.	0	43	100
Internal medicine	33	35.11	24	25.5	24	25.5	8	8.51	4	4.26	1	1.1	94	100
Oncology	4	26.67	7	46.7	3	20	.	0	1	6.67	.	0	15	100
Others	7	46.67	4	26.7	3	20	.	0	1	6.67	.	0	15	100
Total	151	50.5	53	17.7	49	16.4	32	10.7	12	4.01	2	0.7	299	100

Variable	Test	*P*-value	
Speciality	Fisher	0.05	

Dynam (Dynamometry), TUG (“Timed Up and Go”), Chair (Chair test: squats), SPPB (Short Physical Performance Battery), Gait (gait speed test).

In parallel, the participants were asked whether they measured malnutrition in their patients as part of the routine measurements before using the SD algorithm. A total of 73% of the participants indicated that they performed these measurements, with geriatricians doing so most frequently and oncologists the least.

To understand the time required for implementation in clinical practice, participants were asked how long it took them to use the algorithm of malnutrition and DS. Forty-eight percent reported spending less than 5 min to use the algorithm, 40% between 5–7 min, 10% between 8–10 min, and 2% more than 10 min. Overall, 88% of the participants took less than 8 min. Differences were observed between specialities, with oncology having the highest proportion of respondents reporting that it took more than 10 min.

Regarding whether using the malnutrition and DS algorithm has changed their approach to clinical decision-making for nutrition and sarcopenia in their patients with T2DM, 70% of participants agreed or strongly agreed with this statement, with no statistically significant differences detected between specialities.

As part of the management of patients with malnutrition and SD, 97% of participants recommended oral nutritional supplements and 81% recommended physical activity. No statistically significant differences were found between groups, except for referrals to a dietitian for nutritional education or counselling, where endocrinologists were the most likely to recommend this and geriatricians the least likely.

### Consensus questionnaire

All the questions reached consensus in the first round. Table 4 shows the results of the consensus questionnaire.

**Table 4:** Analysis of the consensus questionnaire.

Round 1	Disagreement (1–9)			Indeterminate (4–6)			Agreement (7–9)			Consensus Yes/No
All patients diagnosed with malnutrition should be screened for sarcopenia.									Median 9 (IQR 0)	Yes
A single clinical symptom or a factor raising suspicion is sufficient to screen a person with diabetes for sarcopenia.									Median 9 (IQR 1)	Yes
A duration of diabetes mellitus of 3 years or more should be a key criterion for identifying patients at risk of sarcopenia and/or malnutrition.								Median 8 (IQR 2)		Yes
The diabetic sarcopenia algorithm should be performed in all patients with diabetes.								Median 8 (IQR 3)		Yes
Current levels of blood glucose control should not be a factor in deciding whether to screen a patient for sarcopenic diabetes.							Median 7 (IQR 3)			Yes
Patients who have recently been hospitalised, undergone major surgery or experienced prolonged immobilisation should be prioritised in screening for diabetic sarcopenia.									Median 9 (IQR 1)	Yes
Patients diagnosed with cancer should be prioritised in the screening for diabetic sarcopenia.									Median 9 (IQR 1)	Yes
Screening should be repeated annually in patients at high risk but without a confirmed diagnosis.								Median 8 (IQR 2)		Yes
The frequency of screening for diabetic sarcopenia should be adjusted based on diabetes progression and the presence of complications.									Median 9 (IQR 1)	Yes
A nutritional intervention tailored to the patient’s individual characteristics should be a mandatory part of care following a diagnosis of diabetic sarcopenia.									Median 9 (IQR 1)	Yes
All patients with diabetes who are at risk of sarcopenia should undergo a nutritional assessment at the same time as their muscle assessment.									Median 9 (IQR 1)	Yes
A positive diagnosis of diabetic sarcopenia should be taken into account when determining the patient’s drug treatment.									Median 9 (IQR 1)	Yes
Physical exercise adapted to the patient’s abilities should be a mandatory intervention after the diagnosis of diabetic sarcopenia.									Median 9 (IQR 0)	Yes
The use of severity stages (I, IIa, IIb, IIc) facilitates the classification and management of diabetic sarcopenia.								Median 8 (IQR 2)		Yes
Diabetes-specific oral nutritional supplements containing protein and β-Hydroxy-β-methylbutyrate (HMB) are an essential recommendation for patients with diabetic sarcopenia.								Median 8 (IQR 2)		Yes
I believe the algorithm for the screening, diagnosis and staging of sarcopenic diabetes is suitable for assessing muscle function in people with diabetes.								Median 8 (IQR 1)		Yes

IQR: Interquartile range.

#### Screening

Consensus was reached with a high degree of agreement (8–9/9) that: (a) Patients diagnosed with malnutrition should undergo screening for sarcopenia; (b) All patients with diabetes at risk of sarcopenia should undergo a nutritional assessment alongside a muscle assessment; (c) A clinical symptom or suspicious factor is sufficient to warrant screening for sarcopenia in people with diabetes; (d) A duration of diabetes mellitus of 3 years or more should be considered a risk factor for sarcopenia and/or malnutrition; (e) The DS algorithm should be applied to all patients with diabetes; (f) Screening should be prioritised in patients with recent hospitalisations, major surgery, a diagnosis of cancer, or prolonged immobilisation; (g) The frequency of DS screening should be adjusted according to the progression of diabetes and the presence of complications; (h) It should be repeated annually in high-risk patients without a confirmed diagnosis; (i) Current blood glucose control levels should not be considered when screening for DS in patients with diabetes (7/9). This final question was the item with the lowest agreement scores in the entire questionnaire (median 7 and IQR 3).

#### Nutritional treatment

Consensus was reached with a high degree of agreement (8–9/9) that: (a) In patients with DS, the drug treatment they receive must be considered; (b) Following a diagnosis of DS, a nutritional intervention tailored to the patient’s characteristics must be implemented; (c) In patients who do not meet their nutritional requirements through diet alone, specific oral nutritional supplements for diabetics with a high protein content, including muscle-targeted metabolites such as β-Hydroxy-β-methylbutyrate (HMB), are an essential recommendation; (d) Physical exercise adapted to the patient’s capabilities should be a mandatory intervention. However, it must be noted that this statement reflects expert consensus and indirect evidence extrapolated from general sarcopenia and malnutrition studies, rather than established RCT-level evidence specifically in diabetic sarcopenia populations.

#### Staging

Consensus was reached with a high degree of agreement (8–9/9) that: (a) The use of severity stages (I, IIa, IIb, IIc) facilitates the classification and management of DS.

#### The algorithm

It was agreed, with a high degree of consensus (8/9), that the algorithm for the screening, diagnosis and staging of DS is appropriate for the assessment of muscle function in people with diabetes.

### Usability questionnaire

Figure 1 shows the results of the SUS scale. The results on the scale indicated high usability, with a mean score of 80.34 ± 13.49 points. More than 60% of the participants rated the tool’s usability as excellent. No statistically significant differences were found in SUS scores (0–100) across specialities.

**Figure 1:**
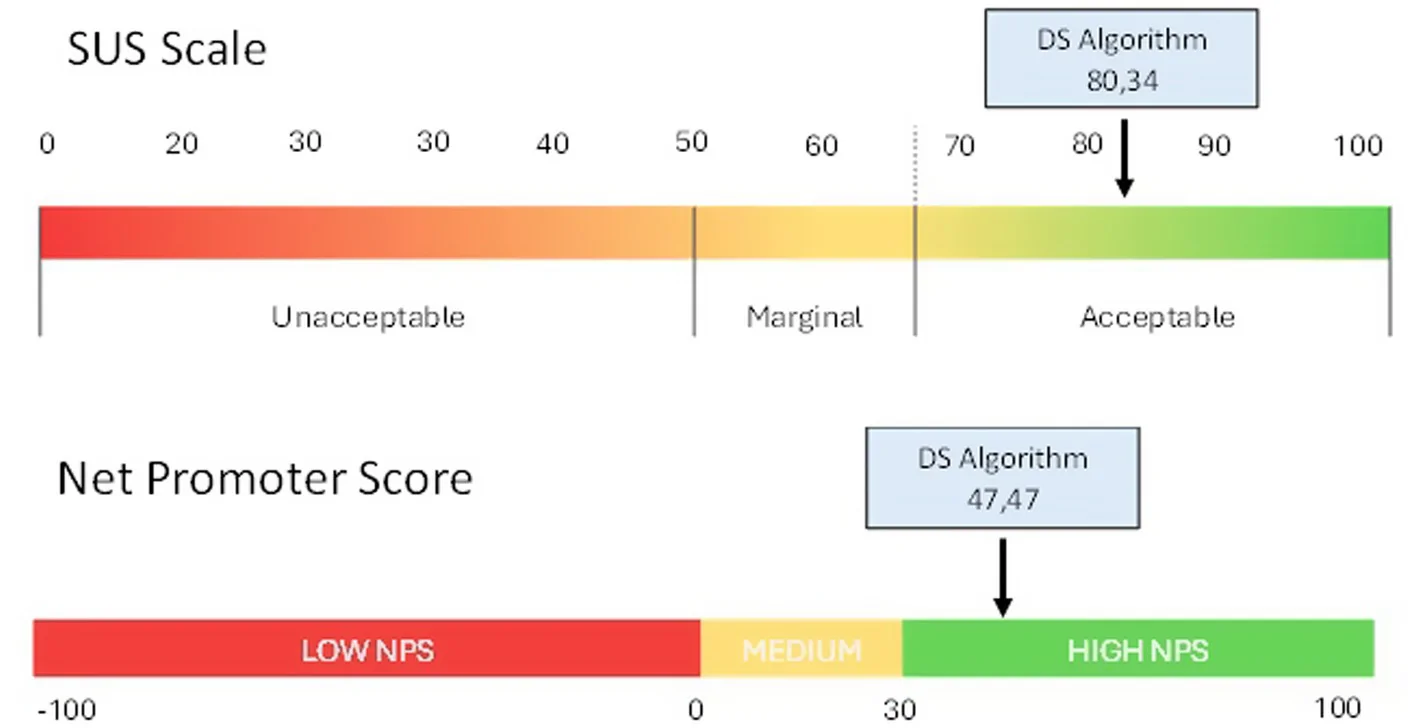
SUS and net promoter scores.

Eighty-six percent of the participants indicated that they would like to use this tool frequently; 81% disagreed with the statement that it is unnecessarily complex; and 90% indicated that it was easy to use. In turn, 70% affirmed that expert support was not needed to use the tool; 87% believed its functions to be well integrated; 87% considered that it did not have many contradictions; 76% did not find it tedious to use; 84% felt confident using the tool; and finally 61% indicated that it was not necessary to know a great deal before being able to start using this tool.

### Healthcare professionals’ feedback questionnaire (net promoter score, NPS)

The probability of recommending the screening algorithm for malnutrition and diabetic sarcopenia to a colleague was 8.6 points on a scale from 1 to 10. The Net Promoter Score, described in the methodology section, was 47.47 (95% CI 40.74–54.21), which is considered a very good result (scores above 50 are excellent). Overall, 53% were promoters and 5% were detractors. No differences were detected according to speciality.

## Discussion

The prevalence of sarcopenia is higher in people with diabetes than in those without diabetes, and it is often underdiagnosed (23). The development of sarcopenia is multifactorial in nature, and involves different pathophysiological mechanisms, notably age-related low-grade chronic inflammation, mitochondrial dysfunction, oxidative stress, hormonal imbalances and alterations in the synthesis and degradation of muscle proteins. Moreover, metabolic conditions such as obesity and diabetes mellitus, characterised by a marked inflammatory component, are closely related to the onset and progression of sarcopenia (24). This, in turn, leads to a vicious cycle of sarcopenia, inflammation, and worsening diabetes.

The present study proposes an algorithm for the screening of risk factors, and for the diagnosis and staging of DS, that has reached a high degree of consensus among specialists in Spain. The study demonstrates the tool’s high usability and a marked willingness among professionals to recommend its implementation in clinical practice. In addition, the integration of complementary information, such as nutritional and muscle assessments, together with the targeted nutritional interventions and physical activity tailored to the patient, supports a more comprehensive clinical approach to affected individuals.

Despite the existence of international consensus guidelines such as EWGSOP2, which establish general criteria, there is a marked lack of screening tools specifically designed for the pathophysiology and phenotype of diabetic patients, who often present with sarcopenic obesity or loss of muscle quality without evident weight loss (9, 25). There is extensive literature on sarcopenia and diabetes, including studies showing the relationship between glycated haemoglobin levels and complications associated with the onset of sarcopenia (26). Many studies have highlighted the importance of a diagnosis of sarcopenia in patients with diabetes, particularly in Asian populations (27–33). Although the clinical framework for this algorithm was established in a previously published expert consensus document by our research group (11), the present study introduces its unified application and constitutes the first formal evaluation of its clinical usability and practical applicability among a large sample of specialists under real-world clinical conditions.

In this context, our study aims to help fill this gap by proposing a specific algorithm, whose usability has been evaluated in clinical practice and which, furthermore, incorporates the consensus of specialists on key aspects of its application.

One of the most cited barriers to the implementation of sarcopenia guidelines is the lack of time and the complexity of traditional diagnostic methods in clinical settings (34). The results of this study show that the proposed algorithm has the potential to address these barriers by providing a rapid and user-friendly too the majority believe the tool is easy to use, does not require expert staff, and 88% take a maximum of 7 min to apply it. This finding is consistent with the urgent need for point-of-care tools which, like the SARC-F questionnaire, allow for rapid screening (35), but with the added advantage in the case of our algorithm of integrating diagnosis and staging into a single workflow. The conclusions of the study indicate that the algorithm is easy to apply and use, thus facilitating its widespread adoption in high-pressure clinical settings.

### Risk stratification and patient prioritisation

Since universal screening may not be feasible in all settings, the Delphi consensus has helped to identify high-risk phenotypes. A notable finding from the questionnaire is that most respondents believe that screening for sarcopenia should be prioritised in patients with a history of recent hospitalisation, major surgery, prolonged immobilisation and malignancies. These clinical red flags align with the evidence that points to hospitalisation and inactivity as potent accelerators of muscle catabolism in patients with pre-existing anabolic resistance (36). The importance of sarcopenia should not be underestimated, and screening should be repeated annually in patients at high risk but without a confirmed diagnosis, reinforcing the view of sarcopenia as a dynamic process that requires continuous monitoring.

Importantly, a significant finding of the study is that most doctors surveyed (70%), reported that the use of the algorithm has changed the approach to decision-making regarding sarcopenia in patients with type 2 diabetes. However, this finding must be interpreted with caution and explicitly contextualized as a self-reported perception subject to selection bias, given that the study cohort consisted of active adopters who had utilized the tool at least five times prior to participation. While this suggests a perceived transition from a purely glucocentric model to a functional one among experienced users, objective evidence of actual practice change and clinical benefit remains to be established. This suggests a transition from a glucocentric model to one focused on functionality. This change is reflected in the therapeutic consensus: physical exercise adapted to the patient’s capabilities should be a mandatory intervention after the diagnosis of DS, and diabetic-specific oral nutritional supplementation including specific protein and muscle protector reached broad agreement as part of a multimodal approach (37). While the physiological actions of HMB including anti-inflammatory, anabolic, and anticatabolic effects are well documented in clinical literature, it must be emphasized that this recommendation relies primarily on expert opinion and extrapolation from clinical trials in general sarcopenia, frail older adults, and disease-related malnutrition. The use of HMB has been well described regarding its anti-inflammatory (38), anabolic (39), and anticatabolic actions (40). However, the direct evidence supporting the use of beta-hydroxy-beta-methylbutyrate (HMB) specifically in patients with diabetic sarcopenia remains scarce (37, 41), and adequately powered randomized controlled trials in this population are needed before firm clinical recommendations can be established. The literature supports this multimodal approach, showing that isolated intervention is less effective than combined exercise and specialised nutritional support for reversing muscle atrophy (42, 43). However, it should be noted that much of the previous data on diet and exercise has been obtained from obese patients rather than older adults, which necessitates the collection of current clinical data from older, non-obese patients with type 2 diabetes—a niche that this algorithm helps to identify.

When screening patients, the duration of having diabetes was reduced from 5 to 3 years as a factor to consider when screening patients, and the current blood glucose control figures should not be a determining factor in deciding whether to screen a patient for DS. The relationship between metabolic control and muscle health has gained critical relevance in recent years, positioning glucose fluctuations as a determinant factor in the development of sarcopenia. Unlike traditional markers, current evidence suggests that daily blood glucose instability has a negative impact on muscle mass and strength, even when glycated haemoglobin (HbA1c) levels are well controlled (44). This independent association underscores that glycaemic variability and impaired pancreatic cell function are indicators of the risk of deterioration in muscle mass and strength (45).

However, the available evidence on the role of HbA1c and its fluctuations in relation to sarcopenia is inconsistent. Some reviews suggest that a HbA1c value ≥ 8.5% is associated with an increased risk of sarcopenia; however, this marker may not adequately reflect daily blood glucose fluctuations, which also appear to play a significant role (46, 47). In contrast, other studies have found no significant association between blood glucose levels and the risk of sarcopenia (48). This heterogeneity of results highlights the need to broaden the clinical approach beyond the measurement of average blood glucose levels, incorporating more dynamic monitoring strategies that prioritise glycaemic stability as a potentially relevant factor in preserving muscle function in patients with diabetes.

A key methodological advantage of this algorithm is the usability validation by a broad range of clinical specialities. Although some heterogeneity in baseline practices was observed – such as differences in the use of GLIM criteria or functional assessments based on specialty, the survey results suggest that the algorithm is appropriate for the cross-sectional assessment of muscle function in people with diabetes. The Net Promoter Score (NPS) and user loyalty indicate that the tool covers an unmet need, standardising clinical language among specialities that traditionally address malnutrition in a compartmentalised manner (17).

### Strengths and limitations

This study benefits from the large size and representativeness of the sample, and that all participants had used the algorithm repeatedly prior to completing the questionnaire, thereby providing a solid basis for assessing its usability under conditions of real-world clinical practice. In addition, the questionnaire addressed a wide range of aspects related to the implementation of the algorithm, including screening, diagnosis, staging, and clinical management criteria, allowing for a comprehensive exploration of potential challenges with its use. Finally, the integration of complementary clinical information into the algorithm, such as nutritional and muscle assessment and the proposal of patient-tailored nutritional and physical activity interventions, supports a multidimensional approach to addressing DS.

Several limitations of this study should be acknowledged. First, as a survey-based descriptive study, the results mainly reflect the perceptions and experiences of the participants, which introduces a certain degree of subjectivity inherent to this type of design. Secondly, a potential selection bias must be acknowledged, as the study focused specifically on healthcare professionals who had used the algorithm in their clinical practice at least five times prior to participating. This inclusion criterion was a deliberate methodological choice to ensure that the usability and consensus evaluations were based on informed professional judgment and actual clinical experience, rather than superficial impressions. While this strategy provides high-quality feedback from experienced users, it inherently excludes the perspectives of clinicians who may have declined to adopt the tool or who encountered early implementation difficulties. Consequently, the high satisfaction and usability scores (SUS and NPS) may reflect a more favorable assessment characteristic of active adopters and might not be fully generalizable to professionals who found the algorithm’s integration into their routine practice more challenging. In addition, the majority of participants represented medical specialists including endocrinology and nutrition, internal medicine and geriatrics, it may limit the generalisation of the results to other clinical settings where patients with diabetes are also treated, such as primary care or other specialities related to the management of frailty and sarcopenia. Another limitation is that all participants were Spanish physicians; therefore, the findings may primarily reflect clinical practice patterns within the Spanish healthcare system. The availability of diagnostic tools, structured nutritional care pathways, and access to multidisciplinary teams may differ substantially across countries and healthcare settings. Consequently, the generalizability of these results to other healthcare systems should be interpreted with caution.

Although this study provides evidence on the acceptability and usability of the algorithm in clinical practice, further research is warranted to assess its impact on relevant clinical outcomes such as improved early detection of sarcopenia, optimisation of patients’ nutritional and functional management and, ultimately, the clinical and functional progression of people with diabetes and a risk of sarcopenia.

## Conclusion

This Delpi-consensus study highlights key recommendations for the screening and management of DS. Most participants supported lower the diabetes duration threshold to 3 years, endorsed the concurrent muscle assessment alongside nutritional assessment; and that, prioritised screening for sarcopenia should be given to patients with a history of recent hospitalisation, major surgery, prolonged immobilisation and malignancies. The importance of sarcopenia should not be underestimated, and screening should be repeated annually in patients at high risk but without a confirmed diagnosis. Physical exercise adapted to the patient’s capabilities should be a mandatory intervention following a diagnosis of DS, and specific oral nutritional supplementation for diabetics containing protein and β-Hydroxy β-methylbutyrate (HMB) appears to be widely accepted.

Overall, the high usability and acceptability ratings, support the suitability of the algorithm for sarcopenia assessment in people with diabetes and it’s use in routine clinical practice by a multidisciplinary healthcare professionals, providing a structured and pragmatic approach to the identification and management of DS. While these results support its suitability for routine clinical practice, further longitudinal studies are needed to confirm its impact on diagnostic accuracy and patient clinical outcomes.

## Data Availability

The raw data supporting the conclusions of this article will be made available by the authors, without undue reservation.
